# Crystal structure of a bis-4-aza­tetra­cyclo[5.3.2.0^2,6^.0^8,10^]dodec-11-ene-3,5-dione compound

**DOI:** 10.1107/S2056989025003500

**Published:** 2025-04-24

**Authors:** Christina Yu Jiang, Richard J. Staples, Shannon M. Biros

**Affiliations:** a Department of Chemistry Grand Valley State University,Allendale MI 49401 USA; b Center for Crystallographic Research, Department of Chemistry and Chemical Biology, Michigan State University, East Lansing, MI 48824, USA; Universidad de la Repüblica, Uruguay

**Keywords:** crystal structure, C-H⋯O hydrogen bonds, C=O⋯π inter­action, imide, [2.2.2]cyclo­octene

## Abstract

In the molecule of 4-({3-[(3,5-dioxo-4-aza­tetra­cyclo­[5.3.2.0^2,6^.0^8,10^]dodec-11-en-4-yl)meth­yl]phen­yl}meth­yl)-4-aza­tetra­cyclo­[5.3.2.0^2,6^.0^8,10^]dodec-11-ene-3,5-dione, C_30_H_28_N_2_O_4_, which contains two substituted [2.2.2]bi­cyclo­octene ring systems linked through a *m*-xylenedi­amine ring, the six-membered rings of the bi­cyclo­octene ring systems adopt nearly perfect boat conformations as determined from Cremer–Pople analysis. Both ring systems are fused to a five-membered imide ring that is oriented *endo* to a bridgehead cyclo­propyl ring. The crystal structure features C=O⋯π inter­actions along with C—H⋯O hydrogen bonds.

## Abstract 2

The crystal structure of the title compound features C=O⋯π inter­actions along with C—H⋯O hydrogen bonds.

## 1. Chemical context

**Figure 1:**
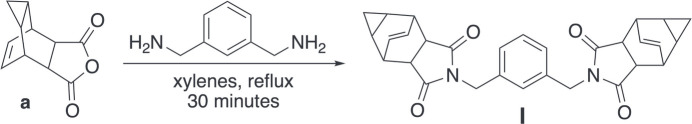
Reaction of anhydride (**a**) and *m*-xylenedi­amine to give compound **I**.

The upper-level synthetic organic laboratory course at Grand Valley State University (GVSU) has exploited the chemistry of compound **a** for its ease of preparation (Kohler *et al.*, 1939 ▸; Kurtz & Johnson, 1989 ▸), readily inter­pretable ^1^H, ^13^C, COSY and HSQC NMR spectra, and its reactivity with primary amines (Fig. 1 ▸). The resulting imide products happen to be quite crystalline and we have reported the structures of three of these compounds in this journal (Hulsman *et al.*, 2020 ▸; Bajko *et al.*, 2024 ▸). Anhydride **a** has been used as a scaffold for the preparation of many new compounds, with one in partic­ular earning approval as a treatment for smallpox (Tecovirimat; Bailey *et al.*, 2007 ▸; Hughes 2019 ▸). We report here the crystal structure of a *bis*-imide derived from anhydride **a** where the [2.2.2]cyclo­octene ring systems have been linked through a *meta*-xylenedi­amine core.
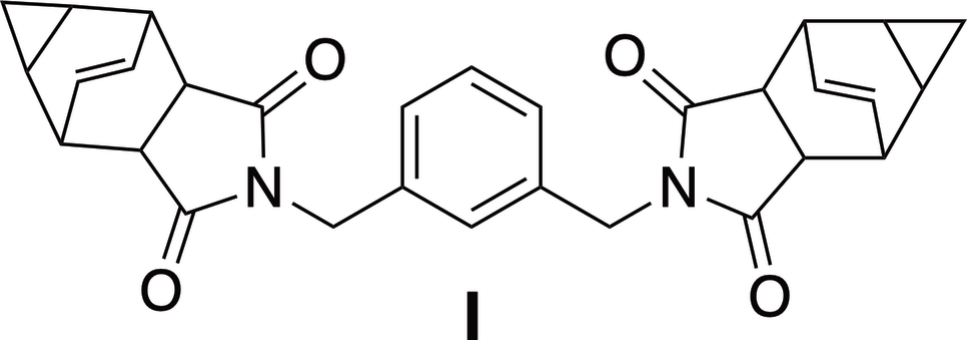


## 2. Structural commentary

**Figure 2:**
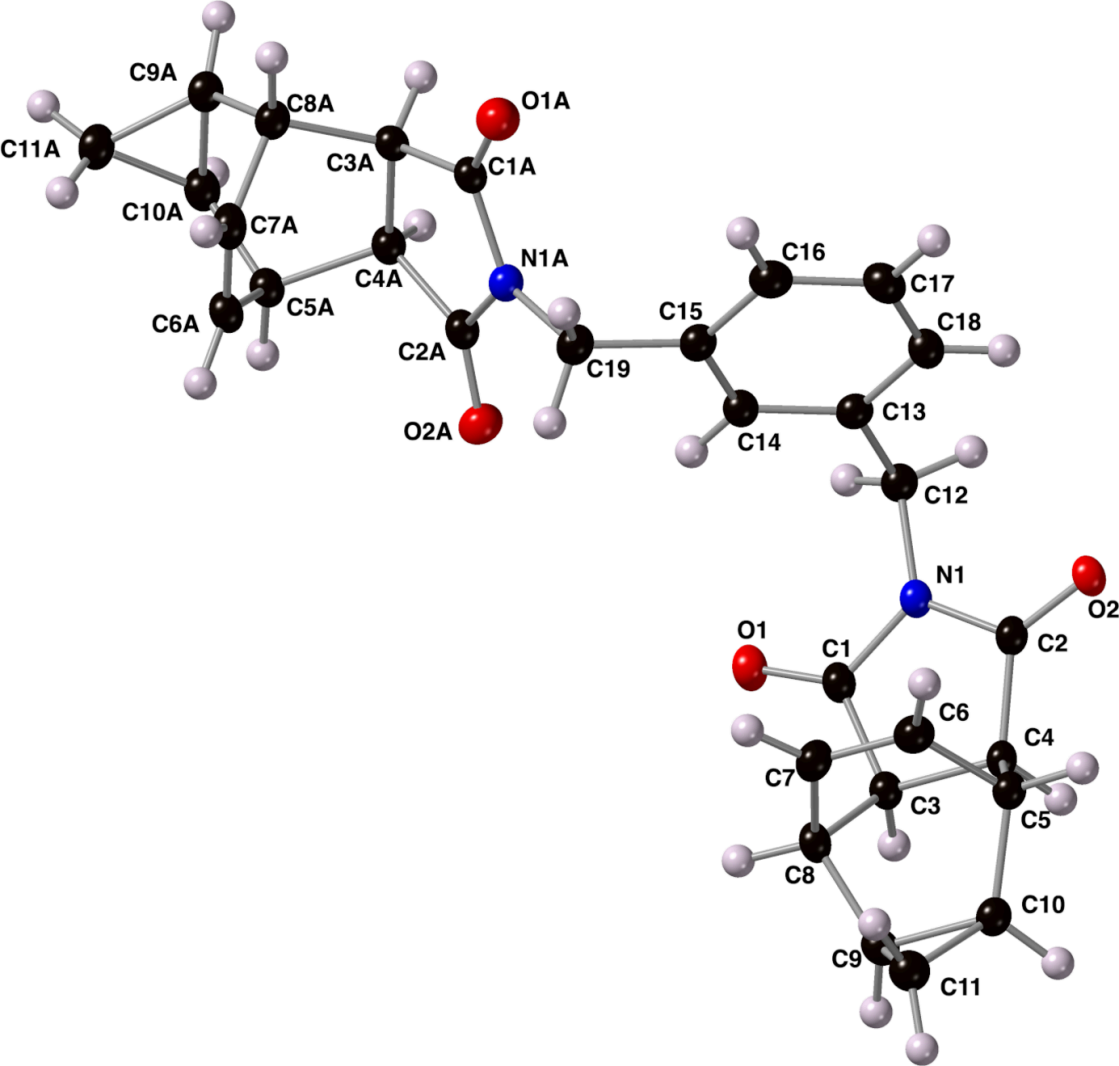
The mol­ecular structure of compound **I** along with the atom-numbering scheme. Displacement ellipsoids are shown at the 50% probability level using standard CPK colors.

The structure of compound **I** is shown in Fig. 2 ▸ along with the atom-numbering scheme. The *meta*-methyl­ene-substituted benzene ring (C13-C18) displays two structurally identical, yet crystallographically unique, 4-aza­tetra­cyclo­[5.3.2.0^2,6^.0^8,10^]dodec-11-ene-3,5-dione ring systems. The bi­cyclo­[2.2.2]cyclo­octene ring systems within each tetra­cycle feature C=C bonds with distances of 1.333 (2) and 1.330 (2) Å for C6—C7 and C6*A*—C7*A*, respectively. The cyclo­hexene rings (C3—C8 and C3*A*—C8*A*) both adopt a nearly perfect boat conformation with Cremer–Pople puckering parameters of 89.89 (10) and 90.82 (11)° for φ and 299.84 (10) and 298.70 (11)° for θ (where φ = 90° and θ = 0° is an ideal boat; Cremer & Pople, 1975 ▸). The imide rings (N1/C1/C3/C4/C2 and N1*A/*C1*A/*C3*A/*C4*A/*C2*A*) are oriented *endo* relative to the bridgehead carbons C9/C10 and C9*A*/C10*A*. The tetra­cyclic ring systems are oriented in nearly opposite directions relative to the planar benzene ring with a N1—C12—C19—N1*A* torsion angle of 135.89 (10)°.

## 3. Supra­molecular features

**Figure 3:**
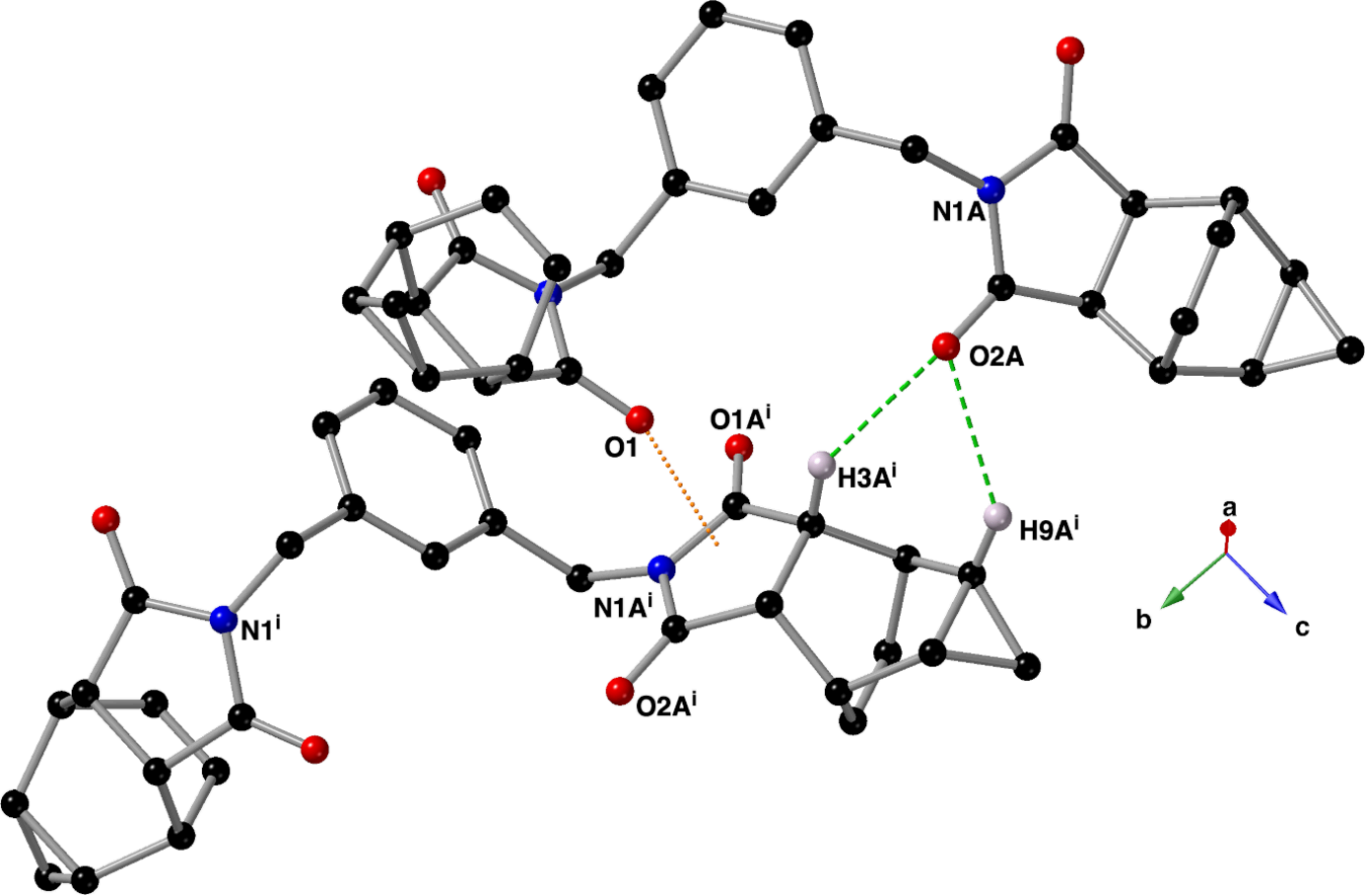
Depiction of the C=O⋯π inter­action (orange, dotted line) and C—H⋯O hydrogen bonds (green, dashed lines) that form dimers of compound **I** in the solid state. This figure was drawn using a ball-and-stick model with standard CPK colors; only hydrogen atoms that are involved in a hydrogen bond are shown for clarity. Symmetry code: (i) −*x* + 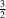, *y* + 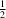, *z*.

**Figure 4:**
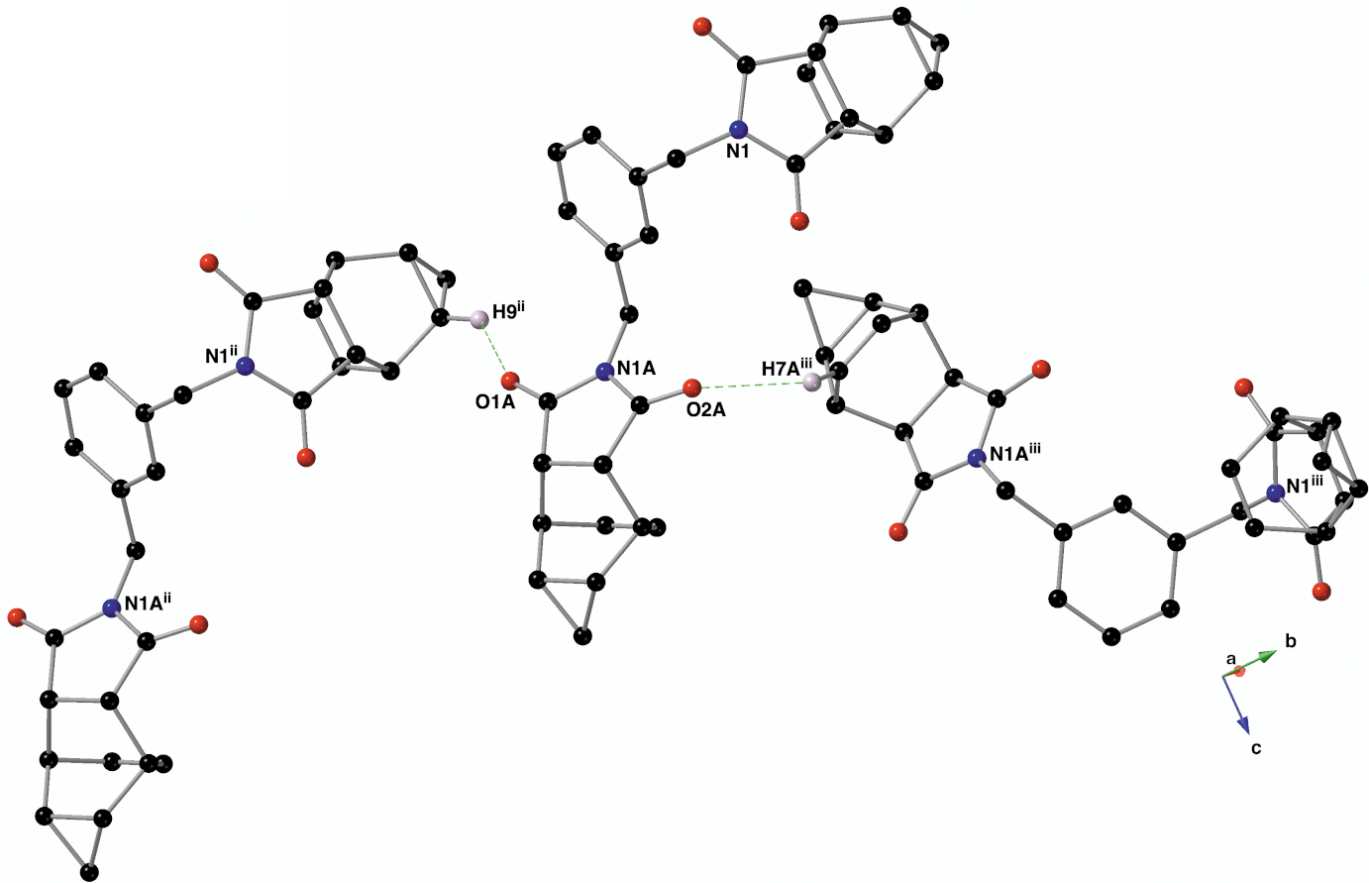
The two additional inter­molecular C—H⋯O hydrogen bonds (green, dashed lines) that are present in the crystal of compound **I** using a standard CPK colors and a ball-and-stick model. Only those hydrogen atoms are shown that are involved in a depicted hydrogen bond. Symmetry codes: (ii) *x*, *y* − 1, *z*; (iii) −*x* + 1, *y* + 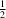, −*z* + 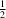.

Dimers of compound **I** are held together through one C=O⋯π inter­action (Mooibroek *et al.*, 2008 ▸; Li *et al.*, 2019 ▸) and two C—H⋯O hydrogen bonds (Fig. 3 ▸; Sutor, 1962 ▸, 1963 ▸; Steiner, 1996 ▸). The C=O⋯π inter­action C1=O1 and the centroid (*Cg*) of the imide N1*A/*C1*A/*C3*A/*C4*A/*C2*A* ring (symmetry code: −*x* + 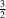, *y* − 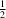, *z*) has an O⋯*Cg* distance of 2.9964 (12) Å with a C=O⋯*Cg* angle of 141.18 (9)°. The C—H⋯O hydrogen bonds are present between C3*A*(H3*A*), C9*A*(H9*A*) and O2*A* (symmetry code: −*x* + 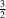, *y* − 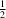, *z*, Table 1 ▸). Each carbonyl oxygen of the N1*A/*C1*A/*C3*A/*C4*A/*C2*A* imide ring hosts an additional C—H⋯O hydrogen bond that links the dimers into sheets that lie in the *ab* plane (Figs. 4 ▸ and 5 ▸). These inter­actions are between atoms C9(H9) and O1*A* (symmetry code: *x*, *y* − 1, *z)* and C7*A*(H7*A*) and O2*A* (symmetry code: −*x* + 1, *y* + 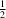, −*z* + 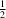).

## 4. Database survey

A search of the Cambridge Structural Database (CSD, version 5.45, updates through June 2024; Groom *et al.*, 2016 ▸) for structures containing a tri­cyclo­[2.2.2.1^4,5^]cyclo­nonene ring system resulted in 31 hits. We highlight here three compounds that we found structurally inter­esting. Structure BOTZIW features four of these tricycles appended to the pyrrole rings of a porphyrin–Pt^II^ complex (Okujima *et al.*, 2019 ▸). In TPCDDD, the tricyclic ring system of the title compound is incorporated into a stunning tri­chloro­penta­cyclo­diene structure (Mock *et al.*, 1972 ▸). Lastly, Kaftory (1978 ▸) crystallized a diadduct formed from two derivatives of the parent tricyclic ring system.

## 5. Synthesis and crystallization

The anhydride shown in Fig. 1 ▸ (205 mg, 1.08 mmol, Kohler *et al.*, 1939 ▸, Kurtz & Johnson, 1989 ▸) was dissolved in 2.0 mL of xylenes in a vial at ambient temperature, then added to a round-bottom flask equipped with a magnetic stir bar. In a separate vial at ambient temperature, 0.1 mL (0.76 mmol) of *m*-xylenedi­amine were dissolved in 1.0 mL of xylenes and then transferred dropwise to the round-bottom flask. A precipitate formed immediately. The reaction mixture was heated to reflux using an oil bath for 30 minutes, allowed to cool to room temperature and diluted with 20 mL of hexa­nes. After standing overnight, the solid was isolated using a Hirsch funnel and recrystallized from hot water. After a few days, orange–yellowish needles appeared in the flask and were isolated. A percentage yield was not determined for this reaction as the amount of product obtained was quite small. Crystals suitable for X-ray diffraction were grown by layering a roughly equal volume of water on top of a dilute sample of the product in DMSO-*d*_6_ in an NMR tube and allowing the solution to sit for two weeks. LR-MS (ESI) *m*/*z*: [*M* + H]^+^ calculated for [C_30_H_28_N_2_O_4_H]^+^ 480.2; found, 480.8.

## 6. Refinement

Crystal data, data collection and structure refinement details are summarized in Table 2 ▸. All hydrogen atoms bonded to carbon atoms were placed in calculated positions and refined as riding: *U*_iso_(H) = 1.2*U*_eq_(C) for methyl­ene, methine, aromatic and alkene groups.

## Supplementary Material

Crystal structure: contains datablock(s) I. DOI: 10.1107/S2056989025003500/ny2012sup1.cif

Structure factors: contains datablock(s) I. DOI: 10.1107/S2056989025003500/ny2012Isup2.hkl

Supporting information file. DOI: 10.1107/S2056989025003500/ny2012Isup3.cml

CCDC reference: 2444823

Additional supporting information:  crystallographic information; 3D view; checkCIF report

## Figures and Tables

**Figure 5:**
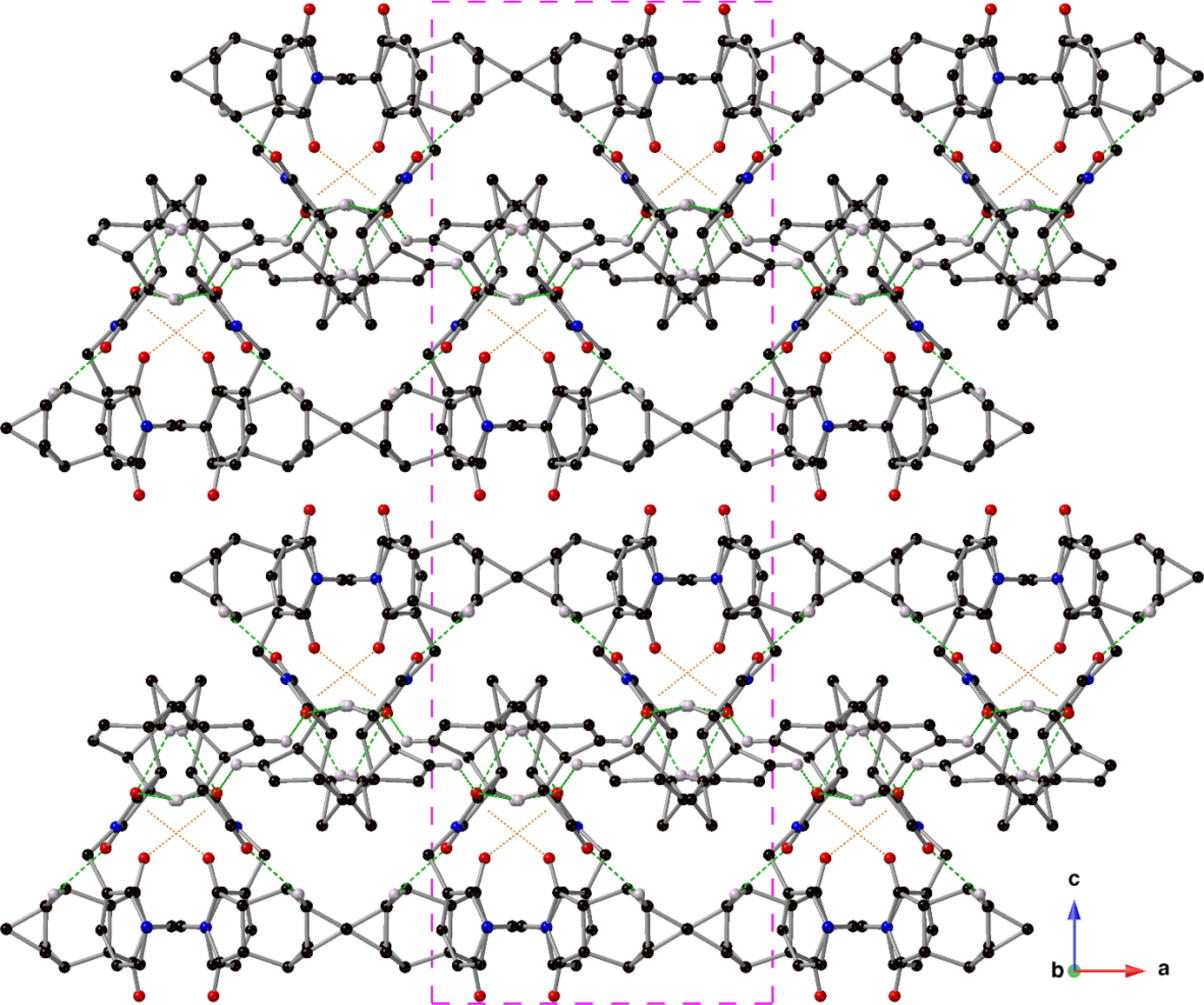
A packing diagram of the crystal of compound **I** viewed down the *b*-axis showing the supra­molecular sheets formed *via* inter­molecular C=O⋯π inter­actions (orange, dotted lines) and C—H⋯O hydrogen bonds (green, dashed lines). Drawn using a ball-and-stick model with standard CPK colors, only hydrogen atoms involved in an inter­action are shown for clarity. The outline of one unit cell is drawn with a pink, dashed line.

**Table 1:** Hydrogen-bond geometry (Å, °)

*D*—H⋯*A*	*D*—H	H⋯*A*	*D*⋯*A*	*D*—H⋯*A*
C3*A*—H3*A*⋯O2*A*^i^	1.00	2.53	3.3958 (17)	144
C9*A*—H9*A*⋯O2*A*^i^	1.00	2.51	3.3041 (17)	136
C9—H9⋯O1*A*^ii^	1.00	2.41	3.1985 (17)	135
C7*A*—H7*A*⋯O2*A*^iii^	0.95	2.52	3.1135 (18)	121

Symmetry codes: (i) 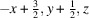; (ii) 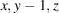; (iii) 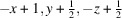.

**Table 2:** Experimental details

Crystal data	
Chemical formula	C_30_H_28_N_2_O_4_
*M*_r_	480.54
Crystal system, space group	Orthorhombic, *Pbca*
Temperature (K)	100
*a*, *b*, *c* (Å)	11.26039 (13), 12.26667 (15), 33.0963 (3)
*V* (Å^3^)	4571.51 (9)
*Z*	8
Radiation type	Cu *K*α
μ (mm^−1^)	0.75
Crystal size (mm)	0.22 × 0.10 × 0.04

Data collection	
Diffractometer	XtaLAB Synergy, Dualflex, HyPix
Absorption correction	Gaussian (*CrysAlis PRO*; Rigaku OD, 2024 ▸)
*T*_min_, *T*_max_	0.824, 1.000
No. of measured, independent and observed [*I* > 2σ(*I*)] reflections	32235, 4946, 4324
*R*_int_	0.045
(sin θ/λ)_max_ (Å^−1^)	0.639

Refinement	
*R*[*F*^2^ > 2σ(*F*^2^)], *wR*(*F*^2^), *S*	0.041, 0.117, 1.07
No. of reflections	4946
No. of parameters	325
H-atom treatment	H-atom parameters constrained
Δρ_max_, Δρ_min_ (e Å^−3^)	0.30, −0.21

Computer programs: *CrysAlis PRO* (Rigaku OD, 2024 ▸), *SHELXT* (Sheldrick, 2015*a* ▸), *SHELXL2019/2* (Sheldrick, 2015*b* ▸), *CrystalMaker* (Palmer, 2007 ▸) and *OLEX2* (Dolomanov *et al.*, 2009 ▸; Bourhis *et al.*, 2015 ▸).

## supplementary crystallographic information

### 4-({3-[(3,5-Dioxo-4-azatetracyclo[5.3.2.02,6.08,10]dodec-11-en-4-yl)methyl]phenyl}methyl)-4-azatetracyclo[5.3.2.02,6.08,10]dodec-11-ene-3,5-dione Crystal data

**Table:**

C_30_H_28_N_2_O_4_	*D*_x_ = 1.396 Mg m^−^^3^
*M**_r_* = 480.54	Cu *K*α radiation, λ = 1.54184 Å
Orthorhombic, *P**b**c**a*	Cell parameters from 15558 reflections
*a* = 11.26039 (13) Å	θ = 2.7–79.2°
*b* = 12.26667 (15) Å	µ = 0.75 mm^−^^1^
*c* = 33.0963 (3) Å	*T* = 100 K
*V* = 4571.51 (9) Å^3^	Needle, colourless
*Z* = 8	0.22 × 0.10 × 0.04 mm
*F*(000) = 2032	

### 4-({3-[(3,5-Dioxo-4-azatetracyclo[5.3.2.02,6.08,10]dodec-11-en-4-yl)methyl]phenyl}methyl)-4-azatetracyclo[5.3.2.02,6.08,10]dodec-11-ene-3,5-dione Data collection

**Table:**

XtaLAB Synergy, Dualflex, HyPix diffractometer	4946 independent reflections
Radiation source: micro-focus sealed X-ray tube, PhotonJet (Cu) X-ray Source	4324 reflections with *I* > 2σ(*I*)
Mirror monochromator	*R*_int_ = 0.045
Detector resolution: 10.0000 pixels mm^-1^	θ_max_ = 80.0°, θ_min_ = 2.7°
ω scans	*h* = −14→14
Absorption correction: gaussian (CrysAlisPro; Rigaku OD, 2024)	*k* = −15→15
*T*_min_ = 0.824, *T*_max_ = 1.000	*l* = −27→42
32235 measured reflections	

### 4-({3-[(3,5-Dioxo-4-azatetracyclo[5.3.2.02,6.08,10]dodec-11-en-4-yl)methyl]phenyl}methyl)-4-azatetracyclo[5.3.2.02,6.08,10]dodec-11-ene-3,5-dione Refinement

**Table:**

Refinement on *F*^2^	0 restraints
Least-squares matrix: full	Hydrogen site location: inferred from neighbouring sites
*R*[*F*^2^ > 2σ(*F*^2^)] = 0.041	H-atom parameters constrained
*wR*(*F*^2^) = 0.117	*w* = 1/[σ^2^(*F*_o_^2^) + (0.056*P*)^2^ + 1.9036*P*] where *P* = (*F*_o_^2^ + 2*F*_c_^2^)/3
*S* = 1.07	(Δ/σ)_max_ < 0.001
4946 reflections	Δρ_max_ = 0.30 e Å^−^^3^
325 parameters	Δρ_min_ = −0.21 e Å^−^^3^

### 4-({3-[(3,5-Dioxo-4-azatetracyclo[5.3.2.02,6.08,10]dodec-11-en-4-yl)methyl]phenyl}methyl)-4-azatetracyclo[5.3.2.02,6.08,10]dodec-11-ene-3,5-dione Special details

**Table:**

Geometry. All esds (except the esd in the dihedral angle between two l.s. planes) are estimated using the full covariance matrix. The cell esds are taken into account individually in the estimation of esds in distances, angles and torsion angles; correlations between esds in cell parameters are only used when they are defined by crystal symmetry. An approximate (isotropic) treatment of cell esds is used for estimating esds involving l.s. planes.

### 4-({3-[(3,5-Dioxo-4-azatetracyclo[5.3.2.02,6.08,10]dodec-11-en-4-yl)methyl]phenyl}methyl)-4-azatetracyclo[5.3.2.02,6.08,10]dodec-11-ene-3,5-dione Fractional atomic coordinates and isotropic or equivalent isotropic displacement parameters (Å^2^)

**Table:**

	*x*	*y*	*z*	*U*_iso_*/*U*_eq_	
O1	0.65542 (9)	0.22161 (9)	0.35506 (3)	0.0280 (2)	
O2	0.64114 (9)	0.24129 (8)	0.49249 (3)	0.0270 (2)	
N1	0.66306 (10)	0.25121 (9)	0.42358 (3)	0.0214 (2)	
C1	0.62872 (12)	0.19453 (11)	0.38916 (4)	0.0214 (3)	
C2	0.62238 (12)	0.20430 (11)	0.45908 (4)	0.0213 (3)	
C3	0.55555 (12)	0.09689 (11)	0.40195 (4)	0.0209 (3)	
H3	0.595702	0.028144	0.393166	0.025*	
C4	0.55262 (12)	0.10284 (10)	0.44859 (4)	0.0203 (3)	
H4	0.592615	0.037261	0.460293	0.024*	
C5	0.42197 (12)	0.10868 (11)	0.46364 (4)	0.0219 (3)	
H5	0.417830	0.115687	0.493710	0.026*	
C6	0.36415 (12)	0.20413 (11)	0.44290 (4)	0.0239 (3)	
H6	0.328886	0.262805	0.457289	0.029*	
C7	0.36653 (12)	0.19948 (11)	0.40267 (4)	0.0248 (3)	
H7	0.333527	0.254937	0.386074	0.030*	
C8	0.42562 (12)	0.09943 (11)	0.38556 (4)	0.0228 (3)	
H8	0.423454	0.098893	0.355357	0.027*	
C9	0.36814 (12)	−0.00344 (11)	0.40349 (4)	0.0243 (3)	
H9	0.390834	−0.074758	0.391057	0.029*	
C10	0.36600 (12)	0.00170 (11)	0.44909 (4)	0.0236 (3)	
H10	0.387699	−0.066222	0.464081	0.028*	
C11	0.25107 (13)	0.00056 (12)	0.42566 (4)	0.0274 (3)	
H11A	0.204812	0.069108	0.424354	0.033*	
H11B	0.202722	−0.066814	0.426447	0.033*	
C12	0.73641 (12)	0.34996 (11)	0.42292 (4)	0.0240 (3)	
H12A	0.785149	0.350056	0.398026	0.029*	
H12B	0.791177	0.348634	0.446316	0.029*	
C13	0.66361 (12)	0.45373 (11)	0.42439 (4)	0.0223 (3)	
C14	0.61529 (12)	0.49699 (11)	0.38902 (4)	0.0226 (3)	
H14	0.628213	0.460844	0.364018	0.027*	
C15	0.54809 (12)	0.59283 (11)	0.38990 (4)	0.0227 (3)	
C16	0.53009 (13)	0.64554 (12)	0.42667 (4)	0.0263 (3)	
H16	0.485926	0.711479	0.427569	0.032*	
C17	0.57654 (14)	0.60192 (12)	0.46204 (4)	0.0294 (3)	
H17	0.562699	0.637477	0.487103	0.035*	
C18	0.64301 (13)	0.50677 (12)	0.46097 (4)	0.0263 (3)	
H18	0.674638	0.477605	0.485287	0.032*	
C19	0.49046 (12)	0.63590 (11)	0.35169 (4)	0.0243 (3)	
H19A	0.451709	0.574514	0.337436	0.029*	
H19B	0.427710	0.688583	0.359291	0.029*	
O1A	0.54311 (10)	0.86502 (8)	0.34502 (3)	0.0302 (2)	
O2A	0.63097 (10)	0.53584 (8)	0.28983 (3)	0.0315 (2)	
N1A	0.57312 (10)	0.68934 (9)	0.32389 (3)	0.0214 (2)	
C1A	0.58996 (12)	0.80106 (11)	0.32221 (4)	0.0219 (3)	
C2A	0.63556 (12)	0.63386 (11)	0.29434 (4)	0.0229 (3)	
C3A	0.67160 (12)	0.82669 (11)	0.28732 (4)	0.0214 (3)	
H3A	0.744287	0.865214	0.297186	0.026*	
C4A	0.70497 (12)	0.71488 (11)	0.26934 (4)	0.0215 (3)	
H4A	0.792121	0.701612	0.272446	0.026*	
C5A	0.66956 (13)	0.71028 (11)	0.22382 (4)	0.0238 (3)	
H5A	0.689249	0.638089	0.211481	0.029*	
C6A	0.53899 (13)	0.73366 (12)	0.22192 (4)	0.0258 (3)	
H6A	0.483456	0.684407	0.210359	0.031*	
C7A	0.50724 (13)	0.82896 (12)	0.23773 (4)	0.0260 (3)	
H7A	0.427000	0.852806	0.238485	0.031*	
C8A	0.60774 (13)	0.89626 (11)	0.25435 (4)	0.0239 (3)	
H8A	0.579303	0.967581	0.265335	0.029*	
C9A	0.70274 (13)	0.91170 (12)	0.22165 (4)	0.0265 (3)	
H9A	0.767960	0.964581	0.227680	0.032*	
C10A	0.73880 (13)	0.80335 (12)	0.20376 (4)	0.0262 (3)	
H10A	0.825524	0.791281	0.199034	0.031*	
C11A	0.67816 (15)	0.88829 (12)	0.17785 (4)	0.0301 (3)	
H11C	0.726822	0.926744	0.157413	0.036*	
H11D	0.594763	0.874818	0.169802	0.036*	

### 4-({3-[(3,5-Dioxo-4-azatetracyclo[5.3.2.02,6.08,10]dodec-11-en-4-yl)methyl]phenyl}methyl)-4-azatetracyclo[5.3.2.02,6.08,10]dodec-11-ene-3,5-dione Atomic displacement parameters (Å^2^)

**Table:**

	*U*^11^	*U*^22^	*U*^33^	*U*^12^	*U*^13^	*U*^23^
O1	0.0300 (5)	0.0369 (6)	0.0171 (4)	−0.0039 (4)	0.0019 (4)	0.0026 (4)
O2	0.0357 (5)	0.0285 (5)	0.0168 (4)	−0.0029 (4)	−0.0039 (4)	−0.0007 (4)
N1	0.0236 (5)	0.0237 (5)	0.0169 (5)	−0.0009 (4)	−0.0013 (4)	0.0019 (4)
C1	0.0208 (6)	0.0259 (6)	0.0174 (6)	0.0022 (5)	−0.0004 (5)	0.0004 (5)
C2	0.0234 (6)	0.0227 (6)	0.0178 (6)	0.0024 (5)	−0.0014 (5)	0.0023 (5)
C3	0.0242 (6)	0.0227 (6)	0.0159 (6)	0.0011 (5)	0.0009 (5)	−0.0004 (4)
C4	0.0252 (6)	0.0207 (6)	0.0151 (5)	0.0014 (5)	−0.0009 (5)	0.0017 (4)
C5	0.0271 (6)	0.0224 (6)	0.0161 (5)	0.0002 (5)	0.0024 (5)	0.0006 (5)
C6	0.0245 (6)	0.0217 (6)	0.0256 (7)	0.0019 (5)	0.0029 (5)	−0.0011 (5)
C7	0.0240 (6)	0.0249 (6)	0.0255 (6)	0.0018 (5)	−0.0015 (5)	0.0059 (5)
C8	0.0258 (6)	0.0278 (7)	0.0149 (5)	−0.0008 (5)	−0.0005 (5)	0.0005 (5)
C9	0.0270 (7)	0.0252 (6)	0.0208 (6)	−0.0026 (5)	−0.0003 (5)	−0.0028 (5)
C10	0.0268 (7)	0.0241 (6)	0.0197 (6)	−0.0016 (5)	0.0011 (5)	0.0024 (5)
C11	0.0265 (7)	0.0298 (7)	0.0257 (6)	−0.0043 (5)	0.0007 (5)	0.0008 (5)
C12	0.0240 (6)	0.0249 (7)	0.0230 (6)	−0.0027 (5)	−0.0015 (5)	0.0025 (5)
C13	0.0220 (6)	0.0227 (6)	0.0221 (6)	−0.0051 (5)	0.0011 (5)	0.0019 (5)
C14	0.0256 (6)	0.0243 (6)	0.0179 (6)	−0.0035 (5)	0.0021 (5)	0.0003 (5)
C15	0.0228 (6)	0.0245 (6)	0.0209 (6)	−0.0046 (5)	0.0025 (5)	0.0026 (5)
C16	0.0272 (7)	0.0242 (7)	0.0275 (7)	−0.0023 (5)	0.0035 (5)	−0.0022 (5)
C17	0.0350 (7)	0.0323 (7)	0.0208 (6)	−0.0054 (6)	0.0040 (5)	−0.0063 (5)
C18	0.0300 (7)	0.0296 (7)	0.0192 (6)	−0.0061 (6)	−0.0006 (5)	0.0013 (5)
C19	0.0233 (6)	0.0263 (6)	0.0234 (6)	−0.0015 (5)	0.0012 (5)	0.0042 (5)
O1A	0.0408 (6)	0.0263 (5)	0.0234 (5)	0.0027 (4)	0.0073 (4)	−0.0045 (4)
O2A	0.0453 (6)	0.0211 (5)	0.0281 (5)	0.0023 (4)	0.0044 (4)	−0.0010 (4)
N1A	0.0245 (5)	0.0213 (5)	0.0182 (5)	0.0002 (4)	0.0012 (4)	0.0006 (4)
C1A	0.0265 (6)	0.0218 (6)	0.0173 (6)	−0.0001 (5)	−0.0032 (5)	−0.0007 (5)
C2A	0.0265 (6)	0.0238 (6)	0.0185 (6)	0.0031 (5)	−0.0024 (5)	−0.0005 (5)
C3A	0.0255 (6)	0.0216 (6)	0.0172 (6)	−0.0013 (5)	−0.0006 (5)	−0.0015 (5)
C4A	0.0227 (6)	0.0235 (6)	0.0182 (6)	0.0021 (5)	−0.0001 (5)	0.0001 (5)
C5A	0.0302 (7)	0.0237 (6)	0.0174 (6)	0.0018 (5)	−0.0004 (5)	−0.0019 (5)
C6A	0.0285 (7)	0.0303 (7)	0.0186 (6)	−0.0033 (5)	−0.0040 (5)	0.0007 (5)
C7A	0.0255 (7)	0.0325 (7)	0.0200 (6)	0.0040 (6)	−0.0016 (5)	0.0037 (5)
C8A	0.0303 (7)	0.0215 (6)	0.0199 (6)	0.0025 (5)	−0.0001 (5)	0.0012 (5)
C9A	0.0331 (7)	0.0271 (7)	0.0193 (6)	−0.0029 (6)	0.0003 (5)	0.0024 (5)
C10A	0.0289 (7)	0.0310 (7)	0.0185 (6)	−0.0001 (6)	0.0013 (5)	0.0004 (5)
C11A	0.0397 (8)	0.0312 (7)	0.0195 (6)	−0.0004 (6)	0.0022 (6)	0.0035 (5)

### 4-({3-[(3,5-Dioxo-4-azatetracyclo[5.3.2.02,6.08,10]dodec-11-en-4-yl)methyl]phenyl}methyl)-4-azatetracyclo[5.3.2.02,6.08,10]dodec-11-ene-3,5-dione Geometric parameters (Å, º)

**Table:**

O1—C1	1.2142 (16)	C15—C19	1.5162 (18)
O2—C2	1.2137 (16)	C16—H16	0.9500
N1—C1	1.3895 (17)	C16—C17	1.389 (2)
N1—C2	1.3861 (16)	C17—H17	0.9500
N1—C12	1.4662 (17)	C17—C18	1.387 (2)
C1—C3	1.5141 (18)	C18—H18	0.9500
C2—C4	1.5122 (18)	C19—H19A	0.9900
C3—H3	1.0000	C19—H19B	0.9900
C3—C4	1.5458 (17)	C19—N1A	1.4640 (17)
C3—C8	1.5605 (18)	O1A—C1A	1.2098 (17)
C4—H4	1.0000	O2A—C2A	1.2127 (17)
C4—C5	1.5548 (18)	N1A—C1A	1.3845 (17)
C5—H5	1.0000	N1A—C2A	1.3834 (17)
C5—C6	1.5052 (18)	C1A—C3A	1.5093 (18)
C5—C10	1.5334 (18)	C2A—C4A	1.5110 (19)
C6—H6	0.9500	C3A—H3A	1.0000
C6—C7	1.333 (2)	C3A—C4A	1.5415 (18)
C7—H7	0.9500	C3A—C8A	1.5606 (18)
C7—C8	1.5065 (19)	C4A—H4A	1.0000
C8—H8	1.0000	C4A—C5A	1.5596 (17)
C8—C9	1.5373 (19)	C5A—H5A	1.0000
C9—H9	1.0000	C5A—C6A	1.499 (2)
C9—C10	1.5108 (18)	C5A—C10A	1.5336 (19)
C9—C11	1.5095 (19)	C6A—H6A	0.9500
C10—H10	1.0000	C6A—C7A	1.330 (2)
C10—C11	1.509 (2)	C7A—H7A	0.9500
C11—H11A	0.9900	C7A—C8A	1.505 (2)
C11—H11B	0.9900	C8A—H8A	1.0000
C12—H12A	0.9900	C8A—C9A	1.5335 (19)
C12—H12B	0.9900	C9A—H9A	1.0000
C12—C13	1.5149 (19)	C9A—C10A	1.511 (2)
C13—C14	1.3958 (18)	C9A—C11A	1.5035 (18)
C13—C18	1.3938 (19)	C10A—H10A	1.0000
C14—H14	0.9500	C10A—C11A	1.512 (2)
C14—C15	1.3984 (19)	C11A—H11C	0.9900
C15—C16	1.3930 (19)	C11A—H11D	0.9900
			
C1—N1—C12	123.91 (11)	C15—C16—H16	119.9
C2—N1—C1	113.28 (11)	C17—C16—C15	120.18 (14)
C2—N1—C12	122.80 (11)	C17—C16—H16	119.9
O1—C1—N1	123.80 (13)	C16—C17—H17	119.8
O1—C1—C3	127.65 (12)	C18—C17—C16	120.38 (13)
N1—C1—C3	108.55 (10)	C18—C17—H17	119.8
O2—C2—N1	124.02 (12)	C13—C18—H18	119.8
O2—C2—C4	127.39 (12)	C17—C18—C13	120.32 (13)
N1—C2—C4	108.59 (11)	C17—C18—H18	119.8
C1—C3—H3	109.9	C15—C19—H19A	108.7
C1—C3—C4	104.68 (10)	C15—C19—H19B	108.7
C1—C3—C8	113.41 (10)	H19A—C19—H19B	107.6
C4—C3—H3	109.9	N1A—C19—C15	114.09 (11)
C4—C3—C8	109.03 (10)	N1A—C19—H19A	108.7
C8—C3—H3	109.9	N1A—C19—H19B	108.7
C2—C4—C3	104.89 (10)	C1A—N1A—C19	123.74 (11)
C2—C4—H4	109.8	C2A—N1A—C19	123.18 (11)
C2—C4—C5	112.34 (10)	C2A—N1A—C1A	112.90 (11)
C3—C4—H4	109.8	O1A—C1A—N1A	123.87 (13)
C3—C4—C5	110.02 (10)	O1A—C1A—C3A	127.44 (12)
C5—C4—H4	109.8	N1A—C1A—C3A	108.67 (11)
C4—C5—H5	111.5	O2A—C2A—N1A	123.57 (13)
C6—C5—C4	107.40 (10)	O2A—C2A—C4A	127.37 (12)
C6—C5—H5	111.5	N1A—C2A—C4A	109.05 (11)
C6—C5—C10	110.16 (11)	C1A—C3A—H3A	110.3
C10—C5—C4	104.41 (10)	C1A—C3A—C4A	104.98 (10)
C10—C5—H5	111.5	C1A—C3A—C8A	111.61 (11)
C5—C6—H6	122.8	C4A—C3A—H3A	110.3
C7—C6—C5	114.43 (12)	C4A—C3A—C8A	109.21 (10)
C7—C6—H6	122.8	C8A—C3A—H3A	110.3
C6—C7—H7	122.6	C2A—C4A—C3A	104.35 (10)
C6—C7—C8	114.78 (12)	C2A—C4A—H4A	110.1
C8—C7—H7	122.6	C2A—C4A—C5A	111.89 (11)
C3—C8—H8	111.7	C3A—C4A—H4A	110.1
C7—C8—C3	107.44 (11)	C3A—C4A—C5A	110.04 (10)
C7—C8—H8	111.7	C5A—C4A—H4A	110.1
C7—C8—C9	109.73 (11)	C4A—C5A—H5A	111.7
C9—C8—C3	104.14 (10)	C6A—C5A—C4A	106.52 (11)
C9—C8—H8	111.7	C6A—C5A—H5A	111.7
C8—C9—H9	116.8	C6A—C5A—C10A	109.76 (11)
C10—C9—C8	110.98 (11)	C10A—C5A—C4A	105.14 (11)
C10—C9—H9	116.8	C10A—C5A—H5A	111.7
C11—C9—C8	121.91 (12)	C5A—C6A—H6A	122.7
C11—C9—H9	116.8	C7A—C6A—C5A	114.54 (13)
C11—C9—C10	59.94 (9)	C7A—C6A—H6A	122.7
C5—C10—H10	117.2	C6A—C7A—H7A	122.5
C9—C10—C5	110.05 (11)	C6A—C7A—C8A	115.08 (13)
C9—C10—H10	117.2	C8A—C7A—H7A	122.5
C11—C10—C5	121.46 (12)	C3A—C8A—H8A	111.8
C11—C10—C9	59.99 (9)	C7A—C8A—C3A	107.58 (11)
C11—C10—H10	117.2	C7A—C8A—H8A	111.8
C9—C11—H11A	117.8	C7A—C8A—C9A	109.53 (11)
C9—C11—H11B	117.8	C9A—C8A—C3A	103.86 (11)
C10—C11—C9	60.07 (9)	C9A—C8A—H8A	111.8
C10—C11—H11A	117.8	C8A—C9A—H9A	116.8
C10—C11—H11B	117.8	C10A—C9A—C8A	110.82 (11)
H11A—C11—H11B	114.9	C10A—C9A—H9A	116.8
N1—C12—H12A	109.0	C11A—C9A—C8A	121.91 (13)
N1—C12—H12B	109.0	C11A—C9A—H9A	116.8
N1—C12—C13	112.89 (11)	C11A—C9A—C10A	60.24 (9)
H12A—C12—H12B	107.8	C5A—C10A—H10A	117.0
C13—C12—H12A	109.0	C9A—C10A—C5A	110.41 (11)
C13—C12—H12B	109.0	C9A—C10A—H10A	117.0
C14—C13—C12	120.23 (12)	C9A—C10A—C11A	59.65 (9)
C18—C13—C12	120.68 (12)	C11A—C10A—C5A	121.92 (13)
C18—C13—C14	119.09 (13)	C11A—C10A—H10A	117.0
C13—C14—H14	119.6	C9A—C11A—C10A	60.11 (9)
C13—C14—C15	120.87 (12)	C9A—C11A—H11C	117.8
C15—C14—H14	119.6	C9A—C11A—H11D	117.8
C14—C15—C19	120.48 (12)	C10A—C11A—H11C	117.8
C16—C15—C14	119.15 (12)	C10A—C11A—H11D	117.8
C16—C15—C19	120.31 (13)	H11C—C11A—H11D	114.9
			
O1—C1—C3—C4	179.09 (13)	C14—C15—C16—C17	−1.2 (2)
O1—C1—C3—C8	−62.18 (18)	C14—C15—C19—N1A	−76.21 (16)
O2—C2—C4—C3	178.79 (13)	C15—C16—C17—C18	1.2 (2)
O2—C2—C4—C5	59.30 (17)	C15—C19—N1A—C1A	−97.03 (15)
N1—C1—C3—C4	−0.66 (14)	C15—C19—N1A—C2A	88.29 (15)
N1—C1—C3—C8	118.07 (11)	C16—C15—C19—N1A	106.82 (14)
N1—C2—C4—C3	−0.93 (14)	C16—C17—C18—C13	−0.2 (2)
N1—C2—C4—C5	−120.42 (11)	C18—C13—C14—C15	0.7 (2)
N1—C12—C13—C14	−83.59 (15)	C19—C15—C16—C17	175.77 (13)
N1—C12—C13—C18	95.83 (15)	C19—N1A—C1A—O1A	4.3 (2)
C1—N1—C2—O2	−179.17 (13)	C19—N1A—C1A—C3A	−174.23 (11)
C1—N1—C2—C4	0.56 (15)	C19—N1A—C2A—O2A	−2.9 (2)
C1—N1—C12—C13	95.96 (15)	C19—N1A—C2A—C4A	175.83 (11)
C1—C3—C4—C2	0.94 (13)	O1A—C1A—C3A—C4A	179.52 (13)
C1—C3—C4—C5	121.97 (11)	O1A—C1A—C3A—C8A	−62.29 (18)
C1—C3—C8—C7	−61.47 (13)	O2A—C2A—C4A—C3A	176.86 (14)
C1—C3—C8—C9	−177.84 (10)	O2A—C2A—C4A—C5A	57.92 (19)
C2—N1—C1—O1	−179.68 (13)	N1A—C1A—C3A—C4A	−2.04 (14)
C2—N1—C1—C3	0.08 (15)	N1A—C1A—C3A—C8A	116.15 (12)
C2—N1—C12—C13	−84.97 (15)	N1A—C2A—C4A—C3A	−1.85 (14)
C2—C4—C5—C6	61.26 (13)	N1A—C2A—C4A—C5A	−120.80 (12)
C2—C4—C5—C10	178.23 (10)	C1A—N1A—C2A—O2A	−178.14 (13)
C3—C4—C5—C6	−55.19 (13)	C1A—N1A—C2A—C4A	0.63 (15)
C3—C4—C5—C10	61.77 (12)	C1A—C3A—C4A—C2A	2.29 (13)
C3—C8—C9—C10	62.45 (13)	C1A—C3A—C4A—C5A	122.48 (11)
C3—C8—C9—C11	129.29 (12)	C1A—C3A—C8A—C7A	−62.90 (14)
C4—C3—C8—C7	54.72 (13)	C1A—C3A—C8A—C9A	−178.96 (11)
C4—C3—C8—C9	−61.65 (12)	C2A—N1A—C1A—O1A	179.45 (13)
C4—C5—C6—C7	57.67 (15)	C2A—N1A—C1A—C3A	0.93 (15)
C4—C5—C10—C9	−62.34 (13)	C2A—C4A—C5A—C6A	58.52 (14)
C4—C5—C10—C11	−128.70 (12)	C2A—C4A—C5A—C10A	174.98 (11)
C5—C6—C7—C8	0.46 (18)	C3A—C4A—C5A—C6A	−57.00 (14)
C5—C10—C11—C9	96.36 (13)	C3A—C4A—C5A—C10A	59.46 (14)
C6—C5—C10—C9	52.70 (14)	C3A—C8A—C9A—C10A	62.70 (14)
C6—C5—C10—C11	−13.66 (16)	C3A—C8A—C9A—C11A	129.81 (13)
C6—C7—C8—C3	−58.46 (15)	C4A—C3A—C8A—C7A	52.73 (14)
C6—C7—C8—C9	54.16 (16)	C4A—C3A—C8A—C9A	−63.33 (13)
C7—C8—C9—C10	−52.31 (14)	C4A—C5A—C6A—C7A	58.06 (15)
C7—C8—C9—C11	14.54 (17)	C4A—C5A—C10A—C9A	−61.42 (14)
C8—C3—C4—C2	−120.71 (11)	C4A—C5A—C10A—C11A	−127.63 (13)
C8—C3—C4—C5	0.32 (14)	C5A—C6A—C7A—C8A	0.41 (17)
C8—C9—C10—C5	0.10 (15)	C5A—C10A—C11A—C9A	96.39 (14)
C8—C9—C10—C11	115.61 (13)	C6A—C5A—C10A—C9A	52.79 (14)
C8—C9—C11—C10	−97.29 (13)	C6A—C5A—C10A—C11A	−13.42 (17)
C10—C5—C6—C7	−55.47 (16)	C6A—C7A—C8A—C3A	−58.00 (15)
C11—C9—C10—C5	−115.51 (13)	C6A—C7A—C8A—C9A	54.27 (15)
C12—N1—C1—O1	−0.5 (2)	C7A—C8A—C9A—C10A	−51.98 (15)
C12—N1—C1—C3	179.24 (12)	C7A—C8A—C9A—C11A	15.13 (18)
C12—N1—C2—O2	1.7 (2)	C8A—C3A—C4A—C2A	−117.51 (11)
C12—N1—C2—C4	−178.61 (11)	C8A—C3A—C4A—C5A	2.69 (15)
C12—C13—C14—C15	−179.88 (12)	C8A—C9A—C10A—C5A	−0.12 (16)
C12—C13—C18—C17	179.82 (13)	C8A—C9A—C10A—C11A	115.72 (14)
C13—C14—C15—C16	0.3 (2)	C8A—C9A—C11A—C10A	−97.25 (15)
C13—C14—C15—C19	−176.71 (12)	C10A—C5A—C6A—C7A	−55.27 (15)
C14—C13—C18—C17	−0.8 (2)	C11A—C9A—C10A—C5A	−115.84 (13)

### 4-({3-[(3,5-Dioxo-4-azatetracyclo[5.3.2.02,6.08,10]dodec-11-en-4-yl)methyl]phenyl}methyl)-4-azatetracyclo[5.3.2.02,6.08,10]dodec-11-ene-3,5-dione Hydrogen-bond geometry (Å, º)

**Table:**

*D*—H···*A*	*D*—H	H···*A*	*D*···*A*	*D*—H···*A*
C3*A*—H3*A*···O2*A*^i^	1.00	2.53	3.3958 (17)	144
C9*A*—H9*A*···O2*A*^i^	1.00	2.51	3.3041 (17)	136
C9—H9···O1*A*^ii^	1.00	2.41	3.1985 (17)	135
C7*A*—H7*A*···O2*A*^iii^	0.95	2.52	3.1135 (18)	121

Symmetry codes: (i) −*x*+3/2, *y*+1/2, *z*; (ii) *x*, *y*−1, *z*; (iii) −*x*+1, *y*+1/2, −*z*+1/2.
